# Long-term renal and survival outcomes in acute kidney injury patients receiving renal replacement therapy in intensive care

**DOI:** 10.1186/cc14385

**Published:** 2015-03-16

**Authors:** I Elsayed, N Pawley, J Rosser, MJ Heap, GH Mills, A Tridente, AH Raithatha

**Affiliations:** 1 Sheffield Teaching Hospitals, Sheffield, UK; 2 Whiston Hospital, St Helens & Knowsley, UK

## Introduction

Acute kidney injury (AKI) affects 40% of critically ill patients, with UK data reporting 5% needing renal replacement therapy (RRT). Hospital mortality is reported as being up to 60%. We sought to evaluate renal and long-term patient survival outcomes in AKI patients receiving RRT on our ICU.

## Methods

Data were collected from our computerised information system on all AKI patients receiving RRT on our ICU, between October 2008 and October 2013. This included demographics, APACHE II and SOFA scores, modality and dose of RRT and ICU length of stay (LOS). Renal and patient survival at ICU discharge was collected, in addition to outcome data at 28 and 90 days and 12 months. Data were examined using Cox proportional hazard multivariate analysis, with Stata 10.1.

## Results

A total of 620 patients with AKI received RRT on our ICU between October 2008 and October 2013. Sixty-one per cent were males. Median age was 65 years (IQR 54 to 74). Median APACHE II score was 23 (IQR 18 to 27). Median SOFA score was 11 (IQR 8 to 13). Fifty-five per cent were mechanically ventilated. A total of 96.7% received CVVH as the principal RRT modality. Twenty-one per cent received a period of high-volume haemofiltration (HVHF) (80 ml/kg/hour), median LOS was 6 days (IQR 3 to 14). In total, 331 (53.4%) patients recovered their renal function at ICU discharge, whilst 237 (38.2%), 220 (35.4%), and 220 (35.4%) patients did not at 28 and 90 days and 12 months respectively. A total of 414 (66.7%) patients survived to ICU discharge, with 368 (59.3%), 341 (55%) and 308 (49.6%) patients being alive at 28 and 90 days and 12 months respectively. Overall patient survival at the end of follow-up was 43%. Adjusting for age and sex; APACHE II score, SOFA score and use of HVHF were associated with worse patient survival at ICU discharge (HR: 1.07, 95% CI: 1.03 to 1.11, *P *< 0.001, HR: 1.11, 95% CI: 1.03 to 1.19, *P *= 0.006 and HR: 2.27, 95% CI: 1.4 to 3.66, *P *= 0.001, respectively). Adjusting for age and sex; APACHE II score and use of HVHF were associated with worse renal recovery at ICU discharge (HR: 1.06, 95% CI: 1.03 to 1.09, *P *< 0.001 and HR: 1.55, 95% CI: 1.03 to 2.3, *P *= 0.032 respectively). SOFA score did not appear to significantly impact renal recovery (HR: 0.99, 95% CI: 0.94 to 1.04, *P *= 0.81).

## Conclusion

Results from our cohort suggest that, in patients with AKI presenting to ICU for RRT, long-term patient survival is significantly impaired. Renal outcomes are poor with 35% being either dialysis dependent or having severe chronic kidney disease (eGFR <15 ml/ minute), at 1 year from ICU discharge. Our data do not suggest a benefit of using HVHF in AKI patients presenting to ICU for RRT.

